# An Implementation Research Approach to Re-orient Health Supply Chains Toward an Equity Agenda in the COVID-19 Era

**DOI:** 10.5334/aogh.3209

**Published:** 2021-04-23

**Authors:** Miriam F. Frisch, Kirstin Woody Scott, Agnes Binagwaho

**Affiliations:** 1 University of Global Health Equity, Kigali, RW; 2 University of Michigan, Ann Arbor, Michigan, USA

## Abstract

The Covid-19 pandemic has exposed critical inequities in global healthcare supply chains and the need for these systems to be analyzed and reoriented with an equity lens. Implementation research methodology can guide the use of evidence-based interventions to re-orient health supply chains towards equity and optimize health outcomes. Using this approach, private and public sector entities can adapt their strategies to focus not just on efficiency and cost savings but ensuring that vulnerable populations have access to essential medications, vaccines, and supplies. Findings can inform regulations that address supply chain inequities at the global level, strengthen existing systems to fill structural gaps at the national level, and address contextual challenges at the subnational level. This methodology can help account for historical practices from prior health initiatives, identify contemporary barriers and facilitators for positive change, and have applicability to the Covid-19 pandemic and ongoing vaccine distribution efforts. An implementation research approach is critical in equipping health supply chains with a path for more resilient and equitable distribution of necessary supplies, vaccines, and delivery of care.

## Viewpoint

The Covid-19 pandemic has illuminated deep fault lines and inequities in healthcare systems, including chronic weaknesses in health supply chains, that must be addressed [1]. Health supply chains are those systems responsible for getting essential medications and supplies from manufacturers to healthcare providers and patients when needed. We argue that a critical analysis of supply chains is needed to re-orient these systems towards improving health outcomes through sustainable and equitable delivery of essential materials, especially within the context of the Covid-19 pandemic. An implementation research framework can help identify the context and roots of dysfunctionalities within the health supply chains as well as strategies to address these challenges.

Lower- and middle-income countries (LMICs) have historically lacked the resources and influence that higher-income countries (HICs) have had over global supply chains with the Covid-19 pandemic only exacerbating this power imbalance [2]. HICs have the ability to purchase essential commodities at high cost, draw from existing stockpiles, limit exports and increase domestic manufacturing [2]. Meanwhile, LMICs have too often been unable to adapt to supply chain challenges such as limited product, lockdowns, and changing export bans, leaving them without sufficient supplies to optimize the safety and care of providers and patients [3]. These power disparities have direct equity implications and translate into uneven risk between those with greater or lesser access to needed resources (e.g., personal protective equipment for healthcare providers or vaccines) when it comes to combating Covid-19 [1,2,4].

Unfortunately, lessons of supply chain failures or gaps due to power disparities found in prior pandemics (including the Ebola epidemic in West Africa) have not translated into stable fixes [5]. Many of these issues have recurred in the current Covid-19 pandemic, such as the continuing shortages of personal protective equipment [5]. This ongoing inequity illustrates the need for health sector leaders to conduct a thorough analysis of the robustness of their supply chains and engage stakeholders in efforts to address known or predictable weaknesses in global, national, and subnational contexts. Analysis of health supply chains with a focus on the strategies used and outcomes achieved when carrying out interventions is key to documenting lessons learned and building more resilient health systems to better respond to future health shocks globally.

In addition to analyzing the gaps, both public and private sector health supply chains need to orient toward an equity-focused agenda to mitigate the long-term disparities evidenced by the Covid-19 pandemic. Strengths and weaknesses within these systems should be identified, leveraged, and addressed to restructure these systems with a focus on health outcomes. Private and public sector manufacturers and distributors of essential goods have historically focused on improving efficiency to maximize profit and cost savings [6]. Yet, these systems should do more to adapt their strategies to local contexts to ensure that vulnerable populations have access to essential medications, vaccines, and supplies.

### Implementation Research as an Approach to Address Supply Chain Inequities

Implementation research can guide the use of evidence-based interventions to re-orient health supply chains towards an equity focus even amidst an ongoing pandemic straining our global health system. Implementation research has been defined as “the scientific study of the use of strategies to adopt and integrate evidence-based health interventions into clinical and community settings to improve individual outcomes and benefit population health” [7]. This methodology provides a mechanism for assessing contextual factors that need to be addressed when implementing evidence-based interventions to achieve improved health outcomes [7].

Within healthcare, evidence-based interventions can include health activities such as community nutrition education sessions, prenatal check-ups, or facility-based deliveries of babies [8]. Within the supply chain context, evidence-based interventions could include the procurement and distribution of vaccines or personal protective equipment. The implementation research frameworks described below can be leveraged to understand why health supply chains have failed in some settings more than others through the understanding of contextual factors, implementation strategies, and implementation outcomes [9,10].

1. *Contextual factors* are defined as “the set of circumstances or unique factors that surround a particular implementation event” [11]. In implementation research frameworks, contextual factors are understood to impact evidence-based interventions. In successful implementation, strategies should be adjusted to accommodate for these factors [8]. Contextual factors that could facilitate successful supply chains include: strong partnerships between governments, donor institutions, manufacturers, access to seaports, and extensive road networks to ease transport and delivery of goods. Limiting contextual factors could include: poor health care facility infrastructure, unreliable electricity for cold storage of vaccines, challenging geographical terrain, and inability to access quality medicine due to resource constraints. Further, corruption and a lack of leadership at national and subnational levels can limit the ability to carry out evidence-based supply chain interventions.
2. *Implementation strategies* are understood as the approaches used for successful and sustained application of interventions [9]. These strategies should be adapted based on the contextual factors that either limit or facilitate an intervention’s ideal implementation [8]. For example, countries can leverage existing donor and community partnerships to aid with Covid-19 vaccine procurement and distribution. To address limitations due to challenging geography or unreliable cold chain storage, vaccines could be delivered via motorbike, rather than car, and involve community leaders to organize single-day vaccination events and identify optimal-transport routes.
3. *Implementation outcomes* are understood as the larger scale results of implementation of evidence-based interventions [10]. Key outcomes related to supply chain initiatives include: acceptability, appropriateness, sustainability, efficiency, effectiveness, equity, and a patient-centered focus [10]. Supply chain related evidence-based interventions should be planned with the goal of meeting key health related implementation outcomes beyond efficient delivery of goods and materials. For example, a patient-centered outcome could adjust implementation strategies to prioritize vaccine delivery to the most vulnerable such as those in rural areas, rather than those with easiest access in urban areas.

However, there are limitations to applying this implementation research approach framework to a supply-chain context. This paper highlighted only a few of many research frameworks that could be applied to this context. Further adapting equity-oriented frameworks to analyze supply chain evidence-based interventions would require that both private and public sector stakeholders have some understanding of contextual factors, strategies, and outcomes. This analysis will require interdisciplinary collaboration, including stakeholder engagement from implementation science experts, health-sector leaders, and supply chain experts from private sector manufacturers to local transporters. During a dynamic pandemic that is already overstretching current capacity in LMICs, facilitating this analysis among key stakeholders and applying it to the Covid-19 pandemic may be challenging. Nonetheless, this approach is crucial in identifying lessons learned and addressing structural challenges within the field.

### Applying an implementation science approach to supply chain challenges

By using an implementation research approach, supply chain evidence-based interventions can be better designed and implemented to link manufacturing of essential materials in factories worldwide to individuals receiving care and protection, especially within LMICs. Here, we provide an example of how implementation research methods have been applied to international supply chain challenges in access to antiretrovirals (ARVs) at all levels of implementation, from the global to subnational levels.

In the mid 2000s, there was a major global effort to achieve universal access to HIV treatment. However, there were distinct barriers for LMICs to meet this objective given the difficulty for them to procure ARVs [12]. At the global level, organizations including Clinton Health Access Initiative (CHAI) and Global Fund to Fight AIDS, Tuberculosis and Malaria (Global Fund) identified complicated supply chains and high costs of procurement as barriers for effective distribution of ARVs to LMICs [12]. They addressed these barriers using strategies including pooled procurement across multiple countries, price negotiations for generic ARVs, and differential pricing based on a country’s HIV burden [12]. They also addressed barriers in high cost manufacturing by providing technical assistance to suppliers to identify cost cutting measures [12]. As a result of interventions carried out at all levels, LMICs were able to secure lower prices for generic ARVs and increase access significantly to vulnerable populations in need of services [12].

At the *national level*, countries faced challenges with forecasting demand and weaknesses in their national logistics systems affecting their ability to distribute ARVs. In Rwanda, the government addressed these barriers by leveraging funding for HIV programs to strengthen national logistics systems, train healthcare workers in supply chain management and develop electronic inventory management systems in partnership with the USAID Global Health Supply Chain Program [13]. These systems are now being leveraged in the roll-out of Covid-19 infection prevention and vaccination initiatives [13,14]. Other countries overcame the lack of human resource capacity by developing logistics and supply chain educational curriculums at local universities to train a new generation of supply chain experts and leveraging technical assistance from global non-profit organizations [15].

At the *subnational level*, barriers were identified in the last mile of ARV delivery and storage. In Ghana, Kenya and South Africa, large regional warehouses were built for storage of ARVs and essential materials to reduce local stockouts and facilitate faster transport to areas with high demand [15]. In recognizing that challenging geographical terrain and poor road quality limits vehicular transport to local clinics, geographic information system analysis has been used by some countries to identify optimal routes or others have utilized innovative technology, such as drones for timely delivery of blood products in remote settings [15,16].

In turn, this approach can be applied at all levels when preparing for and implementing Covid-19 vaccine delivery. For example at the global level, pooled procurement efforts such as with the African Union or COVAX can take into account lessons learned using similar methods used by the Global Fund and CHAI [17,18]. At the national level, existing supply chains built to facilitate ARV distribution can be leveraged and expanded on for effective cold chain storage and transport. Finally, at the subnational level, trained staff and existing community-level knowledge on optimal transport routes will be critical in the final mile of vaccine delivery to patients. Using an approach oriented towards equity, not just efficiency, when carrying out the vaccine initiatives is vital to the delivery of vaccines from manufacturer to the arms of vulnerable populations.

### Looking forward: Equitable Supply Chains to Strengthen Health Systems

For the foreseeable future, the continued spread of Covid-19 and inequities in vaccination supply highlight the importance of conducting supply-chain analyses with an equity lens. An implementation research approach can identify creative solutions that can potentially overcome these barriers, not only for the sake of Covid-19 vaccine distribution, but also to address future health threats. This analysis can lead to adaptation of global regulations, policies and context-specific strategies that optimize equity in healthcare delivery.

In the long term, promoting equitable supply chains through implementation research will help facilitate many countries efforts towards Universal Health Coverage [19]. This collaborative, interdisciplinary, evidence-based approach towards mitigating inequities in supply chains will strengthen the health sector and improve the capacity to provide care to all. In turn, it will ensure that no one is left out not only in the current Covid-19 crisis, but beyond.

## References

[1] Figueroa JP, Bottazzi ME, Hotez P, et al. Urgent needs of low-income and middle-income countries for COVID-19 vaccines and therapeutics. The Lancet. 2021; 397(10274): 562–564. DOI: 10.1016/S0140-6736(21)00242-7 PMC7906712 33516284

[2] McMahon DE, Peters GA, Ivers LC, Freeman EE. Global resource shortages during COVID-19: Bad news for low-income countries. Samy AM, ed. PLoS Negl Trop Dis. 2020; 14(7): e0008412. DOI: 10.1371/journal.pntd.0008412 32628664 PMC7337278

[3] Benson E. COVID-19 (coronavirus): Panic buying and its impact on global health supply chains. World Bank Blogs. https://blogs.worldbank.org/health/covid-19-coronavirus-panicbuying-and-its-impact-global-health-supply-chains. Accessed July 21, 2020.

[4] Demombynes G. COVID-19 Age-Mortality Curves Are Flatter in Developing Countries. World Bank. https://openknowledge.worldbank.org/handle/10986/34028. License: CC BY 3.0 IGO.

[5] Hirschhorn L, Smith JD, Frisch MF, Binagwaho A. Integrating implementation science into covid-19 response and recovery. BMJ. Published online 5 14, 2020: m1888. DOI: 10.1136/bmj.m1888 32409332

[6] Iakovou E, White CC, III. How to Build More Secure, Resilient, next-Gen U.S. Supply Chains. Brookings; 2020. https://www.brookings.edu/techstream/how-to-build-more-secure-resilient-next-gen-u-s-supply-chains/.

[7] National Institutes of Health. Dissemination and Implementation Research in Health (R01 Clinical Trial Optional). Published 2019. https://grants.nih.gov/grants/guide/pa-files/PAR-19-274.html

[8] Hirschhorn L, Frisch M, Ntawukuriryayo J, VanderZanden A, Donahoe K, Mathewos K. Development and application of a hybrid implementation research framework to understand success in reducing under-5 mortality in Rwanda. Gates Open Res. (in press) Published online 2021. DOI: 10.12688/gatesopenres.13214.1 PMC8688814 35079696

[9] Proctor EK, Powell BJ, McMillen JC. Implementation strategies: Recommendations for specifying and reporting. Implement Sci. 2013; 8(1): 139. DOI: 10.1186/1748-5908-8-139 24289295 PMC3882890

[10] Proctor E, Silmere H, Raghavan R, et al. Outcomes for implementation research: Conceptual distinctions, measurement challenges, and research agenda. Adm Policy Ment Health Ment Health Serv Res. 2011; 38(2): 65–76. DOI: 10.1007/s10488-010-0319-7 PMC3068522 20957426

[11] Damschroder LJ, Aron DC, Keith RE, Kirsh SR, Alexander JA, Lowery JC. Fostering implementation of health services research findings into practice: A consolidated framework for advancing implementation science. Implement Sci. 2009; 4(1): 50. DOI: 10.1186/1748-5908-4-50 19664226 PMC2736161

[12] Waning B. Global strategies to reduce the price of antiretroviral medicines: Evidence from transactional databases. Bull World Health Organ. 2009; 87(7): 520–528. DOI: 10.2471/BLT.08.058925 19649366 PMC2704041

[13] USAID. Global Health Supply Chain Program: Rwanda. USAID. Accessed June 18, 2020. https://www.ghsupplychain.org/country-profile/rwanda.

[14] APA-Kigali. Rwanda. Rwanda begins COVID-19 vaccine rolllout. http://apanews.net/en/news/rwanda-begins-covid-19-vaccine-rollout.

[15] Pastakia SD, Tran DN, Manji I, Wells C, Kinderknecht K, Ferris R. Building reliable supply chains for noncommunicable disease commodities: Lessons learned from HIV and evidence needs. AIDS. 2018; 32(Supplement 1): S55–S61. DOI: 10.1097/QAD.0000000000001878 29952791

[16] Glauser W. Blood-delivering drones saving lives in Africa and maybe soon in Canada. Can Med Assoc J. 2018; 190(3): E88–E89. DOI: 10.1503/cmaj.109-5541 29358207 PMC5780273

[17] COVAX: Working for equitable access to Covid-19 vaccines. https://www.who.int/initiatives/act-accelerator/covax.

[18] African Union African Supplies Medical Platform. https://amsp.africa/astrazeneca-covid-19-vaccine/.

[19] Binagwaho A, Frisch MF, Udoh K, et al. Implementation research: An efficient and effective tool to accelerate universal health coverage. Int J Health Policy Manag. Published online 2020. DOI: 10.15171/ijhpm.2019.125 PMC7306110 32563218

